# Challenges in Meeting Dose Constraints During Left Postmastectomy Radiotherapy in a Patient With a Leadless Pacemaker

**DOI:** 10.7759/cureus.97855

**Published:** 2025-11-26

**Authors:** Koyo Kikuchi, Ibuki Ota, Hiromori Sasaki, Ryuji Nakamura

**Affiliations:** 1 Radiation Oncology, Iwate Medical University, Yahaba-cho, JPN; 2 Radiation Oncology, Iwate Prefectural Central Hospital, Morioka, JPN; 3 Radiology, Morioka Red Cross Hospital, Morioka, JPN

**Keywords:** breast neoplasms, cardiac implantable electronic devices, cardiac pacing, dose constraints, leadless pacemaker, mastectomy, postmastectomy radiotherapy, radiotherapy complications, radiotherapy planning

## Abstract

A 60-year-old woman with a 20-year history of cardiac pacing presented with node-positive left breast cancer and underwent total mastectomy. Postmastectomy radiotherapy (PMRT) was indicated; however, because a subcutaneous pacemaker was located in the left anterior chest wall, it was replaced with a leadless pacemaker (LPM) to avoid direct irradiation. PMRT was planned using three-dimensional conformal radiotherapy with a conventional regimen of 50 Gy in 25 fractions, targeting the left chest wall, supraclavicular region, and internal mammary nodes, followed by a boost of 10 Gy in five fractions to the enlarged left supraclavicular and internal mammary nodes. Based on published guidelines and the manufacturer’s specifications, our institutional dose constraints were defined as the maximum point dose <200 cGy for the LPM and <500 cGy for the planning risk volume of the LPM (PRV_LPM; LPM plus a 1-cm margin). The initial calculation revealed doses of 314 cGy to the LPM and 565 cGy to the PRV_LPM, exceeding the limits. To reduce the dose to the LPM, the inferior border of the chest wall field was reduced while maintaining coverage of the surgical scar and enlarged left internal mammary nodes. Consequently, the LPM and PRV_LPM doses decreased to 102 cGy and 154 cGy, respectively, achieving compliance with the predefined constraints. This case demonstrates that LPM implantation alone does not necessarily ensure compliance with device dose limits during conventionally fractionated left-sided PMRT.

## Introduction

Cardiac implantable electronic devices (CIEDs), including pacemakers, are susceptible to malfunctions such as electrical resets after radiotherapy [1]. International guidelines recommend minimizing the radiation dose to pacemakers, with threshold limits typically ranging between 200 and 1000 cGy, according to risk stratification [2-7]. Specifically, the 2022 European Society of Cardiology guideline recommends that CIEDs should not be positioned within the treatment volume and sets a cumulative dose limit of ≤200 cGy for pacemakers [2]. Similarly, the American Association of Physicists in Medicine (AAPM) TG-203 report recommends avoiding direct irradiation of the device whenever possible [3].

Postmastectomy radiotherapy (PMRT) for node-positive breast cancer reduces locoregional and distant recurrence, as well as long-term breast cancer mortality, and is therefore recommended by current guidelines for eligible patients [8-10]. Because PMRT generally encompasses both the chest wall and the supraclavicular region, the presence of a pacemaker on the ipsilateral chest frequently requires generator relocation, removal, or meticulous planning to avoid direct exposure.

A leadless pacemaker (LPM) integrates the pulse generator and electrode into a single intracardiac device, eliminating transvenous leads and the need for a subcutaneous generator. LPMs are typically implanted in the right ventricle, and this design may facilitate breast irradiation [11]. However, particularly in patients with cardiomegaly, where the right ventricle lies close to the chest wall, data remain limited on whether the LPM dose can be maintained within the recommended limits in the setting of left-sided PMRT.

We report the case of a patient with LPM who was scheduled to undergo conventionally fractionated PMRT to the chest wall, supraclavicular region, and internal mammary nodes after mastectomy for regionally advanced left-sided breast cancer. This case underscores that, although in clinical practice there may be a tendency to assume that replacing a conventional pacemaker with an LPM ensures the safe delivery of PMRT, it highlights that even with LPM placement, achieving device dose constraints in conventionally fractionated radiotherapy can still be challenging.

## Case presentation

**Figure 1:**
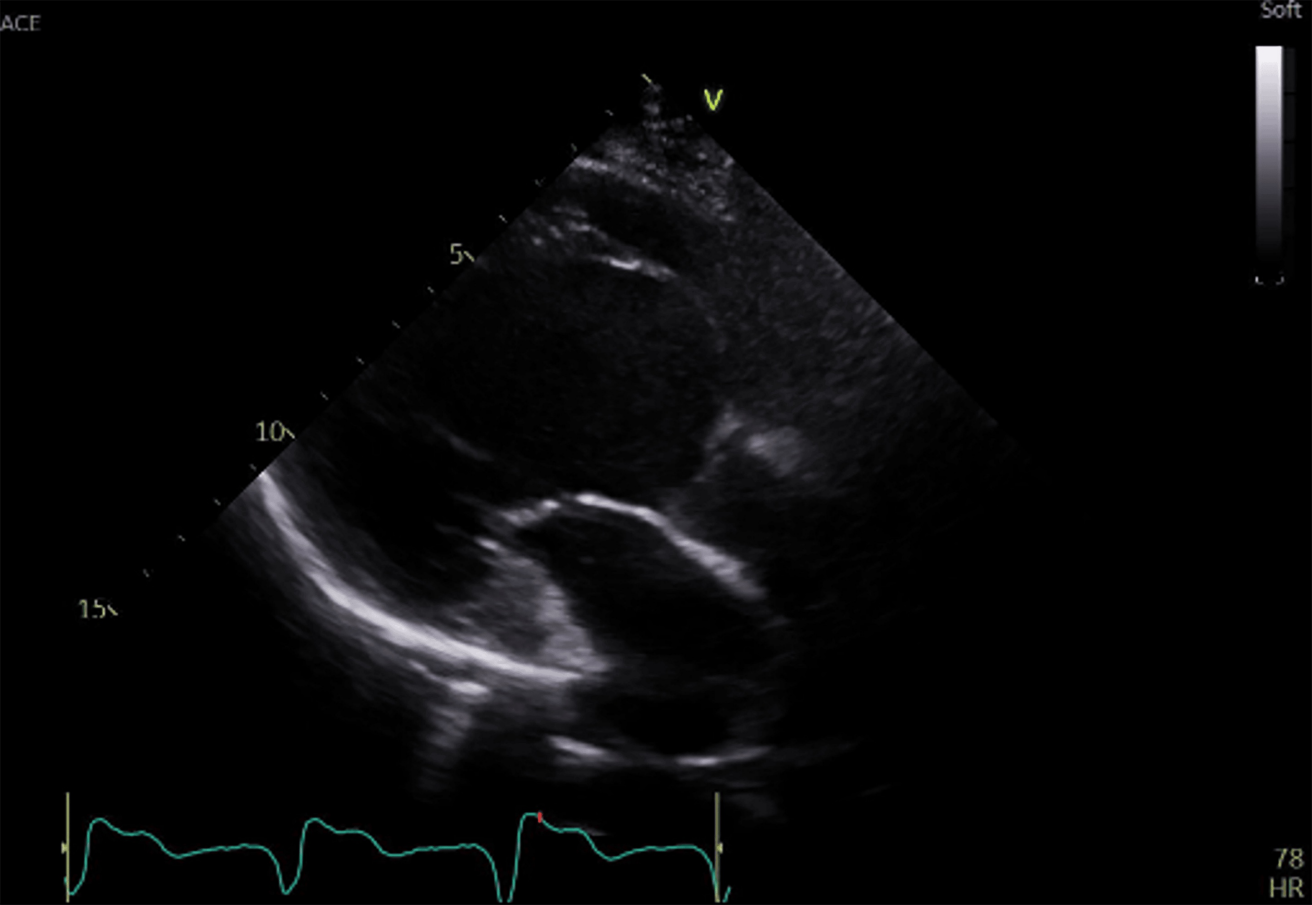
Preoperative echocardiographic findings. Left ventricular systolic dysfunction was observed, with a left ventricular ejection fraction of 14% (Teichholz method).

A 60-year-old woman presented to the Department of Breast Surgery at our hospital. The patient was diagnosed with an invasive ductal carcinoma, HER2-enriched subtype in the upper outer quadrant of her left breast, which was clinically staged as T3N3cM0. The patient also had a 20-year history of complete atrioventricular block and sick sinus syndrome secondary to cardiac sarcoidosis, which was addressed by implanting a pacemaker in the left anterior chest wall. Preoperative evaluation revealed considerable cardiac dysfunction with a left ventricular ejection fraction of 14% (Teichholz method) to 42% (modified Simpson method), akinesis at the base of the heart, and diffuse hypokinesis in the mid-to-apical segments, findings that were interpreted as evidence of impaired left ventricular function (Figure 1). Neoadjuvant chemotherapy was omitted based on the recommendation of the cardiologist. The patient subsequently underwent left total mastectomy with level III axillary dissection and was then referred to the Department of Radiation Oncology for PMRT.

**Figure 2:**
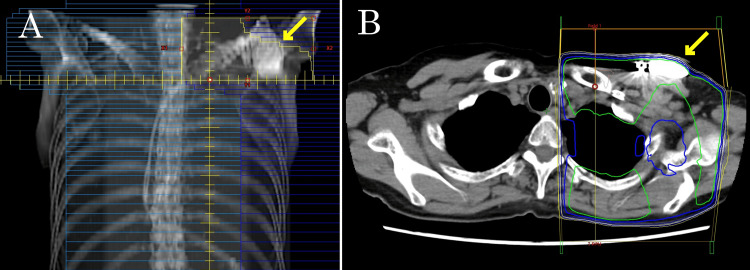
The irradiation field for the left supraclavicular region directly overlaps the pacemaker implanted subcutaneously in the left anterior chest wall. (A) Digitally reconstructed radiograph of the supraclavicular field showing overlap with the pacemaker. (B) CT image with dose distribution showing direct radiation exposure to the pacemaker.

The tumor was pathologically staged as T4bN3aM0. The PMRT fields encompassed the left chest wall, supraclavicular region, and internal mammary nodes. However, the existing pacemaker was located within the supraclavicular field (Figure 2), necessitating consideration of device dose management during treatment planning. Following multidisciplinary evaluation, the cardiology team implanted a LPM (Micra™ AV MC1AVR1; Medtronic, Minneapolis, MN) in the right ventricular septum and subsequently explanted the pulse generator of the existing device, leaving the leads in situ rather than relocating them.

**Figure 3:**
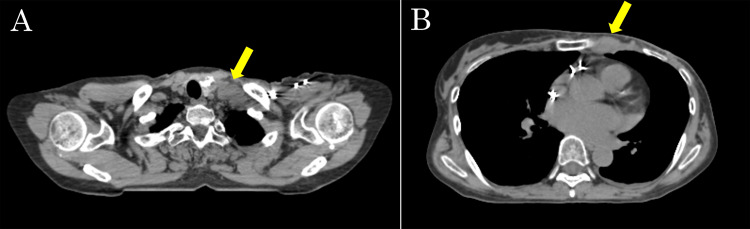
Simulation CT images showing enlarged lymph nodes. (A) Left supraclavicular and (B) left internal mammary lymph nodes are indicated by arrows. These nodes were defined as the nodal gross tumor volume (nGTV).

The external beam radiotherapy system consisted of a computed tomography (CT) simulator (Aquilion LB; Canon Medical Systems, Otawara, Japan), a planning system (Eclipse Version 15.1; Varian Medical Systems, Palo Alto, CA), and a linear accelerator (Clinac iX; Varian Medical Systems, Palo Alto, CA). Simulation CT with 2-mm slices under free breathing identified several enlarged left supraclavicular and internal mammary nodes, defined as the nodal gross tumor volume (nGTV) (Figure 3). Although PMRT with the deep inspiration breath-hold technique was considered, it was ultimately abandoned because the prolonged delivery time was deemed intolerable for a patient with severe cardiac dysfunction, despite its potential advantage of increasing the distance between the heart and chest wall to reduce the LPM dose.

In the 3D conformal plan, the clinical target volume encompassed the left chest wall, the supraclavicular region, and the internal mammary nodes in the third intercostal space. Organs at risk comprised the spinal cord, heart, lungs, LPM, and a 1-cm isotropic margin around the LPM, defined as the planning risk volume of the LPM (PRV_LPM), to account for respiratory and cardiac motion. The prescribed dose consisted of 50 Gy in 25 fractions, followed by a 10 Gy boost to the nGTV in five fractions. Using the half-beam block (mono-isocenter technique), the isocenter was positioned midway between the midline and lateral border of the left scapula at the level of the inferior sternoclavicular joint [12]. To minimize neutron production and reduce the associated risk of LPM malfunction, photon beam energies ≤10 MV were selected, in accordance with AAPM TG-203 [3]. Superior fields were delivered by opposing beams at 345°(4 MV) and 165°(10 MV), while inferior fields were delivered at 300°and 120°(4 MV). For LPM shielding, multileaf collimators were shaped to maintain a 1.5-cm margin from the LPM and 0.5 cm from the PRV_LPM. A 5-mm bolus was applied, and the prescription point for the chest wall field was set to 5 mm beneath the skin.

**Figure 4:**
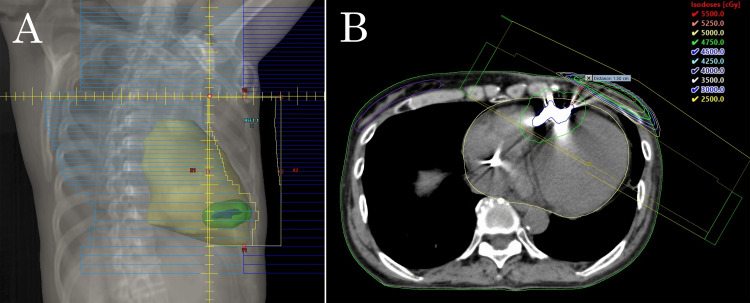
Dose distribution from the initial 3D conformal plan. The heart (yellow), leadless pacemaker (LPM) (blue), planning organ-at-risk volume (PRV_LPM) (green), and right breast (purple) are contoured. (A) Digitally reconstructed radiograph of the chest wall field with a gantry angle of 300°. (B) Multileaf collimators were configured to maintain a 1.5-cm distance from the LPM for shielding, and a 1-cm isotropic margin was applied to define the PRV_LPM. Nevertheless, the LPM received a maximum dose of 314 cGy and the PRV_LPM 565 cGy, both exceeding institutional dose constraints.

**Figure 5:**
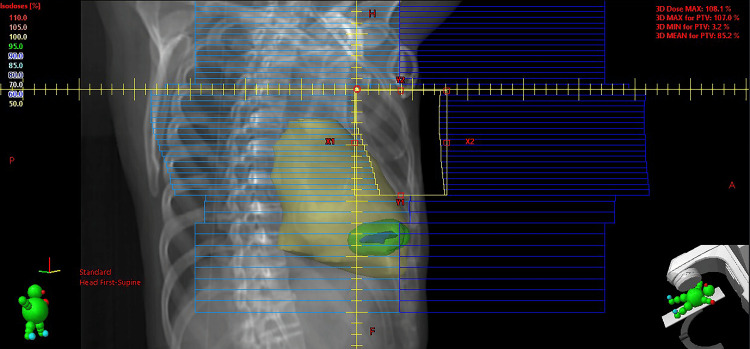
Revised 3D conformal plan with upward adjustment of the lower jaw to meet dose constraints for the leadless pacemaker (LPM). This modification reduced the maximum dose to the LPM to 102 cGy and to the planning organ-at-risk volume (PRV_LPM) to 154 cGy.

Dose calculations in the initial plan (50 Gy in 25 fractions) indicated a maximum dose of 314 cGy to the LPM and 565 cGy to the PRV_LPM, exceeding the institutional constraints (LPM <200 cGy, PRV_LPM <500 cGy) (Figure 4). These constraints were defined by adopting the conservative 200 cGy limit from the guidelines [2] for the device, and the 500 cGy limit from the manufacturer’s specifications [13] for the PRV_LPM. To meet these constraints, the inferior border of the chest wall field was reduced, decreasing the field size from 8.3 × 16.8 to 8.3 × 9.4 cm (Figure 5). This adjustment lowered the maximum doses to the LPM and PRV_LPM to 102 cGy and 154 cGy, respectively. However, dose coverage of the chest wall became suboptimal, and the chest wall volume receiving at least 90% of the prescription dose (V90%) decreased from 76.8% to 60.4%. The chest wall scar and nGTV were included in the modified treatment field.

**Figure 6:**
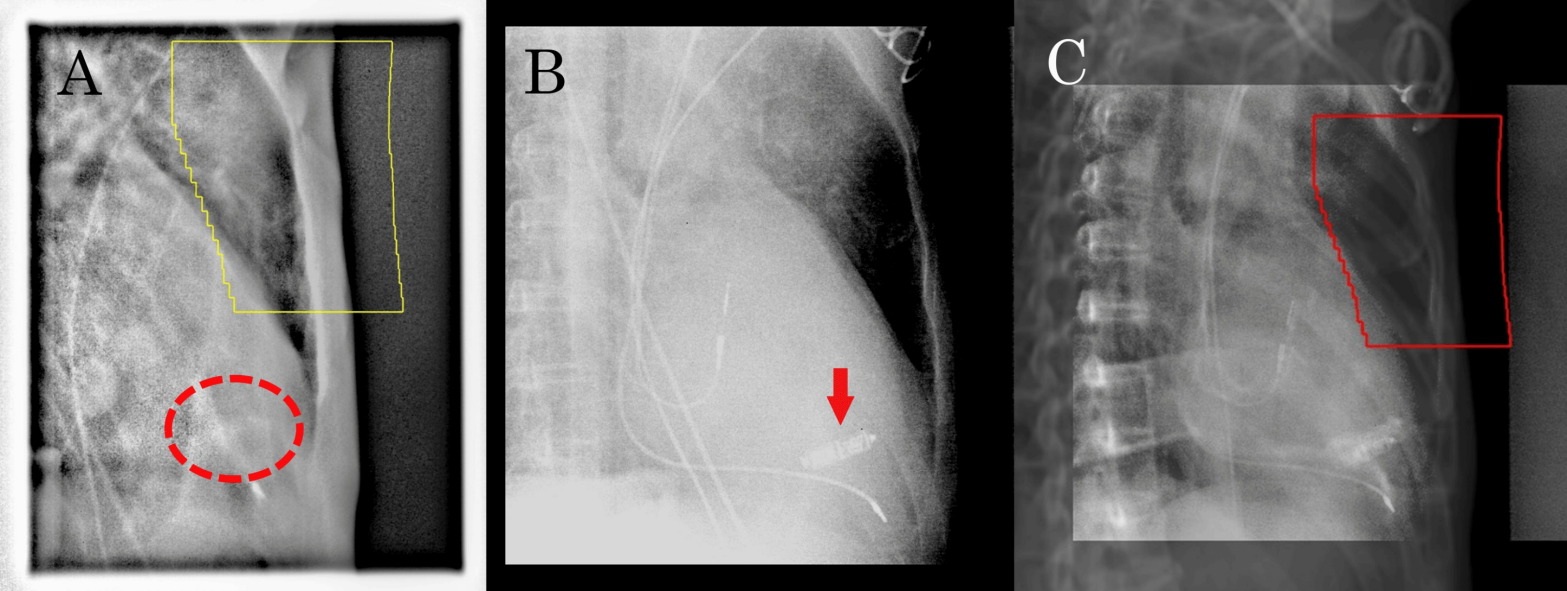
Image-guided verification of pacemaker position (A) Electronic portal imaging on the day before treatment. The expected location of the leadless pacemaker (LPM), outlined by a red dashed circle, is not clearly visible. The yellow contour indicates the radiation field. (B) Kilovoltage setup image on the same day. Residual pacing leads from the previous device are seen. The red arrow indicates the LPM. Using fluoroscopic mode, the LPM was confirmed to move outside the treatment field. (C) Image registration of the digitally reconstructed radiograph and kilovoltage image on the treatment day. The red contour indicates the radiation field.

Pretreatment setup was performed one day before the first fraction, which corresponded to seven weeks after mastectomy. The LPM was not clearly visible on electronic portal imaging but could be identified by its motion on kV fluoroscopy and was confirmed to be outside the field defined by the jaws. On the day of treatment, the kV image-guided setup was verified against a digitally reconstructed radiograph, and the first fraction was delivered uneventfully (Figure 6). The LPM was interrogated before and/or after treatment, and no abnormalities were detected.

Prior to the second fraction, the breast surgeon noted findings on the simulation CT suggestive of liver metastases and recommended initiating systemic therapy with pertuzumab, trastuzumab, and docetaxel rather than continuing PMRT. As a result, only one fraction of radiotherapy was delivered. Despite systemic therapy, the disease progressed, and the patient died of heart failure following duodenal ulcer bleeding 27 months after radiotherapy. No episodes of LPM malfunction were observed during the treatment.

## Discussion

Currently, evidence regarding radiotherapy in patients with LPMs remains limited. Wang et al. replaced a conventional pacemaker with an LPM before whole-breast irradiation using deep-inspiration breath-hold and ultrahypofractionation (26 Gy in 5 fractions plus 10.4 Gy in two fractions), maintaining a device dose <200 cGy [11]. Ultrahypofractionation often yields a lower cumulative dose than conventional fractionation, which may facilitate device dose management. In contrast, PMRT is typically delivered with conventional fractionation (45-50.4 Gy in 25-28 fractions) while moderate hypofractionation (40-42.5 Gy in 15-16 fractions) has more recently been recommended [14]. Ultrahypofractionation is not standard for PMRT, particularly when nodal irradiation is indicated [10,15]. In Japan, moderate hypofractionation is not routinely used for PMRT because of limited evidence and reimbursement constraints.

In the current case, complete coverage of the chest wall was compromised to comply with the device dose constraints. When treatment coverage and device safety are in conflict, individualized clinical judgment is essential. In such situations, we prioritize keeping the device dose within conservative limits and then determine, through a multidisciplinary conference and shared decision making with the patient, whether the resulting reduction in target coverage is acceptable. Munshi et al. reported that shielding a chest wall pacemaker within tangential fields during whole-breast irradiation was acceptable when the partial-breast criteria were met, and the lumpectomy cavity remained fully encompassed [16]. In this case, we prioritized regional nodal irradiation over full chest wall coverage because lymph node metastases were present. We also generated a trial volumetric modulated arc therapy plan targeting the entire chest wall; however, because of leakage from the multileaf collimator, it was difficult to keep the dose to the pacemaker body below 200 cGy.

Saki et al. described a case of bilateral breast cancer treated with proton therapy after LPM implantation. Although the device received an average dose of only approximately 1 cGy (relative biological effectiveness), electrical reset alerts were triggered on treatment days 11 and 27, highlighting a potential risk associated with the neutron-generating modalities [17]. In the present case, 10 MV photons were delivered to the supraclavicular field to optimize dose distribution. Device malfunction rates of up to 10% have been observed with 15-18 MV photons and proton therapy [18]. In addition, Gauter-Fleckenstein et al. reported that, in patients treated with photon energies of 6-23 MV, the relative risk of CIED failure for exposures >6 MV was 9.03 (95% confidence interval: 5.24-15.55) [19]. Whether 10 MV photons constitute a high-risk energy level remains guideline-dependent, with some guidelines classifying it as high-risk [2,4,5], while others do not [3,6,7]. Notably, Medtronic provides no specific recommendations regarding photon energy [13].

Another practical issue is assuming that LPMs are safe solely because they are intraventricular. The proximal edges of the tangential or chest wall fields may approach the right ventricular silhouette, particularly in patients with a chest wall deformity, large breast volume, or cardiac dilation. Therefore, pre-implant planning should include an estimation of the achievable minimum dose to the right ventricular LPM under a realistic PMRT geometry.

## Conclusions

In PMRT, a conventional pacemaker on the ipsilateral chest complicates planning. LPM implantation represents a potential alternative, but adherence to device dose constraints can still be challenging with conventional fractionation. Pretreatment simulation should assess whether LPM replacement is appropriate, and planning should consider breath-hold techniques, appropriate PRV margins, and hypofractionated schedules when clinically feasible. Interdisciplinary coordination and individualized dosimetric assessment remain essential to ensure both oncologic efficacy and cardiac device safety.
